# Clinical neuropathology practice guide 1-2013: Molecular subtyping of glioblastoma: ready for clinical use? 

**DOI:** 10.5414/NP300605

**Published:** 2012-12-27

**Authors:** Adelheid Woehrer, Christine Marosi, Georg Widhalm, Stefan Oberndorfer, Josef Pichler, Johannes A. Hainfellner

**Affiliations:** 1 Institute of Neurology,; 2 Department of Medicine I,; 3 Department of Neurosurgery, Medical University of Vienna, Vienna,; 4 Department of Neurology, State Hospital St. Pölten, St. Pölten,; 5 Internal Medicine and Neurooncology, Landesnervenklinik Wagner-Jauregg, Linz, Austria

**Keywords:** glioblastoma, subtypes, gene-expression, genetic aberrations, epigenetics

## Abstract

Recently, integrated genome-wide analyses have revealed several glioblastoma (GB) subtypes, which differ in terms of key pathogenetic pathways and point to different cells of origin. Even though the proneural and mesenchymal GB signatures evolved as most robust, there is no consensus on the exact number of subtypes and defining criteria. Moreover, important issues concerning within-tumor heterogeneity and class-switching upon recurrence remain to be addressed. Early evidence indicates an association of different GB subtypes with patient outcome and response to therapy, which argues for the implementation of molecular GB subtyping, and consideration of GB subtypes in subsequent patient management. As genome-wide analyses are not routinely available to the majority of neuropathology laboratories, first attempts to implement immunohistochemical testing of surrogate markers are underway. However, so far, confirmatory studies are lacking and there is no consensus on which markers to use. Further, the rationale for testing is compromised from a clinical point of view by a lack of effective therapies for individual GB subtypes. Thus, incorporation of genomic research findings as a basis for GB patient management and clinical decision making currently remains a perspective for the future.

## Rationale of glioblastoma subtypes 

**Figure 1:**
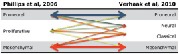
Figure 1. Molecular GB subtypes: gene-expression based hierarchical groupings [7]. Direct comparison across Phillips’ and Verhaak’s datasets shows near complete agreement for proneural and mesenchymal GB signatures, whereas there is less concordance for proliferative and neural/classical GB subtypes [7].

Genome-wide analyses have provided substantial insights into the underlying biology of many cancers [1, 2]. With regard to glioblastoma (GB), comprehensive approaches integrating gene expression, DNA sequencing and copy number data have established several molecular subtypes. Although no consensus exists, 2 – 6 GB subtypes have emerged, which are characterized by distinct gene expression profiles and genetic aberrations [3, 4, 5, 6, 7, 8]. Those differentially expressed genes or “cancer signatures” reflect key routes of pathogenesis, growth characteristics, and divergent differentiation pointing to different cells of origin [5, 6, 7]. Across the various datasets, the mesenchymal and proneural GB signatures are consistently found, whereas there is less concordance for proliferative, neural, and classical GB subtypes, respectively [7]. A comparison between GB subtypes according to Phillips et al. [5] and Verhaak et al. [6] is presented in Figure 1 adapted from Huse et al. [7] For a comprehensive characterization of GB subtypes according to Verhaak et al. [6] see Table 1. 

Subsequent integrated analyses of proteomic markers [9] and methylation data [10] have further expanded and refined the molecular genetic complexity of the disease across all age cohorts [8]. Most importantly, hypermethylation at a large number of CpG islands (glioma-CpG island methylator phenotype (G-CIMP+)) has been found to be associated with proneural GBs (Proneural/G-CIMP+ subtype) [10]. This epigenetic signature is also associated with low-grade gliomas and secondary/recurrent GB. 

## Prognostic and predictive considerations 

Although GBs are considered a single histological entity according to the WHO classification [11], they are molecularly diverse tumors with differences in biologic behavior and response to treatment. However, despite intense efforts only few clinically relevant markers are known so far, including isocitrate dehydrogenase 1 (*IDH1*) mutation status and O-6-methylguanine-DNA methyltransferase (*MGMT*) promoter methylation. The presence of the *IDH1* mutation is a strong positive prognostic marker associated with younger patient age and longer overall survival [12, 13]. However, *IDH1* mutations are prevalent in proneural and secondary GBs, but rare in all other primary GBs [14]. *MGMT* promoter methylation is associated with longer overall survival times through an enhanced response to alkylating drugs, e.g., temozolomide [15], and higher incidence of pseudoprogression [16]. 

Recently, significant differences in prognosis and therapeutic response have similarly been advocated for individual GB subtypes [3, 6]: patients with proneural/G-CIMP+ tumors are on average younger at the time of diagnosis and experience a significantly improved outcome, whereas the mesenchymal signature is associated with an infiltrative behavior, and thus aggressive disease course [6, 10, 17]. However, there is evidence that patients with mesenchymal and classical GB particularly benefit from combined treatment, whereas those with proneural tumors do not [6, 18]. Therefore, testing of GB subtypes in the routine diagnostic setting might be of clinical relevance. 

## Translation of molecular GB subtyping into routine clinical use 

**Figure 2:**
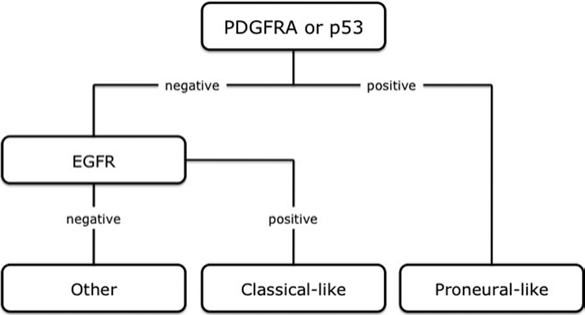
Proposed algorithm for immunohistochemical analyses of GB subtypes according to Le Mercier et al. [18].

Gene-expression and methylation-based studies of GB subtypes are hardly feasible in routine diagnostic neuropathology as they require enormous technical and financial resources, usually necessitate fresh-frozen tissue, and are not generally applicable to individual patients [7, 18]. Hence, several immunohistochemical “surrogate” markers have recently been suggested to distinguish GB subtypes [18, 19]. In contrast to expression- or methylation-based analyses, immunohistochemistry is available to the vast majority of neuropathology laboratories, can be easily conducted on standard formalin-fixed and paraffin-embedded samples, and the evaluation of the protein expression is morphology-controlled. In a recently conducted study, Le Mercier et al. [18] were able to distinguish proneural-like and classical-like GB subtypes based on immunohistochemical analyses of EGFR, PDGFRA, and p53. The proprosed algorithm is shown in Figure 2 [18]. Moreover, in their patient set they confirmed a significantly longer survival of patients with proneural-like GBs. Whereas patients with classical-like tumors showed an increased benefit from combined treatment, this was not evident in the proneural-like cohort. The authors suggested that further markers such as MET, NF1 or YKL-40 could be introduced for assessment of mesenchymal-like GBs [18]. 

## Caveats 

Despite the promising initial characterization of GB subtypes, several caveats need to be taken into account: 

1. So far, there is *no consensus* on the defining criteria and number of GB subtypes. 

2. There are conflicting results on whether or not GB might shift toward the mesenchymal phenotype upon recurrence (“*class switching*”) [5, 10, 20]. 

3. *Within-tumor heterogeneity* might drive variation in gene expression [21, 22] and several expression signatures might be present within the same tumor (communication on occasion of the European Association for Neurooncology (EANO) congress 2012). 

## Summary and clinical performance 

The recent molecular-genetic characterization of GB subtypes has considerably improved our understanding of the disease complexity with regard to differences in key pathogenetic pathways. There is early evidence that the various GB subtypes are associated with differences in prognosis and therapeutic response. In fact, different GB subtypes may require different therapeutic approaches (“personalized medicine”) [5, 6]. However, so far, defining criteria for molecular GB subtypes have not yet been sufficiently settled and caveats like class-switching and within-tumor heterogeneity necessitate further investigations. The rationale for molecular GB subtyping is also compromised from a clinical point of view by the current lack of effective therapies for individual GB subtypes. Thus, incorporation of genomic research findings as a basis for GB patient management and clinical decision making currently remains a perspective for the future. 

## Conflict of interest 

None declared. 

**Table 1:** Characteristics of GB subtypes according to Verhaak et al. [6].

Classical GB	The classical GB subtype shows an expression signature which resembles astroglia and is characterized by frequent chromosome 7 amplifications and chromosome 10 deletions, 95% showing *EGFR* amplification. This class lacks aberrations in *TP53*, *NF1*, *PDGFRA*, or *IDH1* genes.
Mesenchymal GB	The mesenchymal GB subtype is associated with gene expression of angiogenesis and inflammation, reflecting extensive necrosis and prominent inflammatory cell infiltration. Those tumors show a high frequency of neurofibromin *NF1* mutations/deletions, as well as high expression of CHI3L1 and MET.
Proneural GB	The proneural GB subtype is associated with younger age, *PDGFRA* abnormalities, and *IDH1* and *TP53* mutations, which have previously been associated with secondary GB. They might arise from a progenitor or neural stem cell that can also give rise to oligodendrogliomas.
Neural GB	Neural GBs bear the highest resemblance to samples derived from normal brain tissue. Thus, their gene expression profile suggests a cell of origin with a differentiated phenotype.
